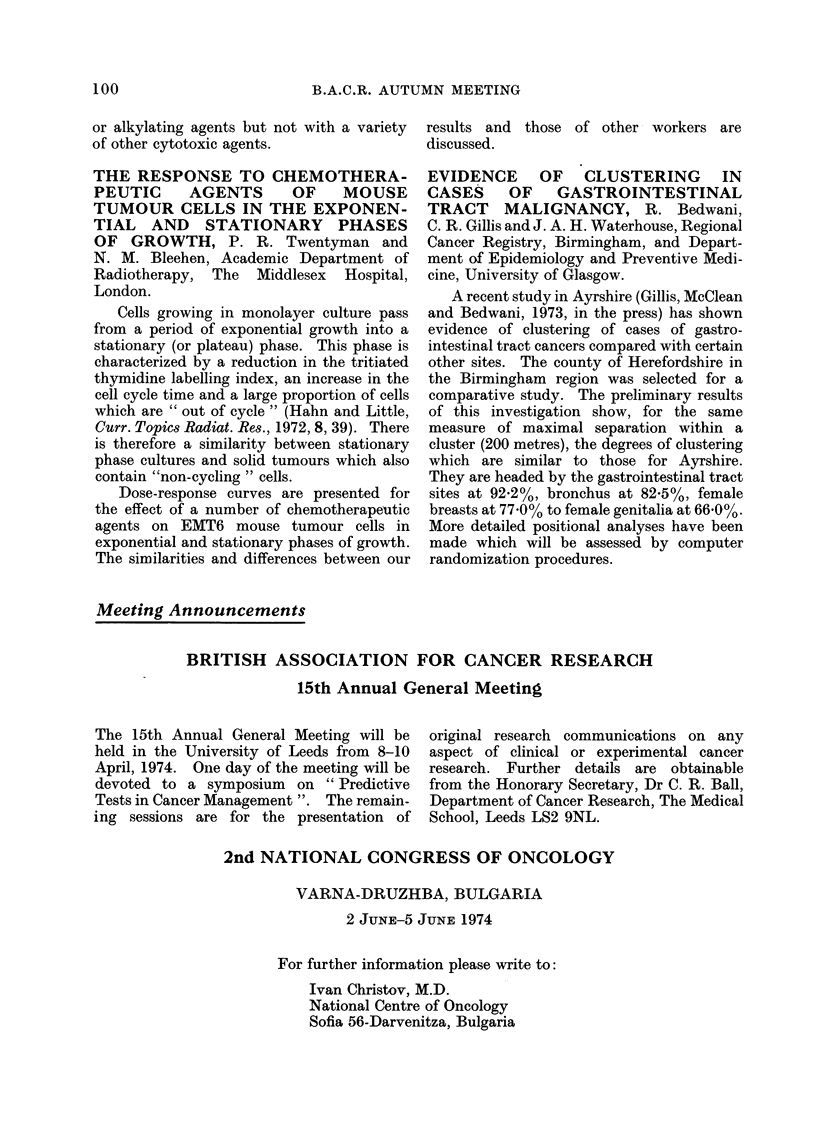# Proceedings: Evidence of clustering in cases of gastrointestinal tract malignancy.

**DOI:** 10.1038/bjc.1974.41

**Published:** 1974-01

**Authors:** R. Bedwani, C. R. Gillis, J. A. Waterhouse


**EVIDENCE OF CLUSTERING IN CASES OF GASTROINTESTINAL TRACT MALIGNANCY,** R. Bedwani, C. R. Gillis and J. A. H. Waterhouse, Regional Cancer Registry, Birmingham, and Department of Epidemiology and Preventive Medicine, University of Glasgow.

A recent study in Ayrshire (Gillis, McClean and Bedwani, 1973, in the press) has shown evidence of clustering of cases of gastrointestinal tract cancers compared with certain other sites. The county of Herefordshire in the Birmingham region was selected for a comparative study. The preliminary results of this investigation show, for the same measure of maximal separation within a cluster (200 metres), the degrees of clustering which are similar to those for Ayrshire. They are headed by the gastrointestinal tract sites at 92·2%, bronchus at 82·5%, female breasts at 77·0% to female genitalia at 66·0%. More detailed positional analyses have been made which will be assessed by computer randomization procedures.